# Phage ImmunoPrecipitation Sequencing (PhIP-Seq): The Promise of High Throughput Serology

**DOI:** 10.3390/pathogens11050568

**Published:** 2022-05-11

**Authors:** Charles Kevin Tiu, Feng Zhu, Lin-Fa Wang, Ruklanthi de Alwis

**Affiliations:** 1 Programme in Emerging Infectious Diseases, Duke-NUS Medical School, Singapore 169857, Singapore; charles.tiu@u.duke.nus.edu (C.K.T.); feng.zhu@duke-nus.edu.sg (F.Z.); linfa.wang@duke-nus.edu.sg (L.-F.W.); 2 SingHealth Duke-NUS Global Health Institute, Singapore 169857, Singapore; 3 Viral Research and Experimental Medicine Centre (ViREMiCS), SingHealth Duke-NUS Academic Medical Centre, Singapore 169856, Singapore

**Keywords:** phage-immunoprecipitation sequencing, PhIP-Seq, sero-epidemiology, phage, antibody, diagnostic, surveillance, immunity, serology, VirScan

## Abstract

**Simple Summary:**

Determining the exposure or infection history of a person to a multitude of viruses is not an easy task. Typically, antibody tests detect antibodies against proteins (antigens) to only one or a few viruses. Here, we review an emerging technology called Phage ImmunoPrecipitation Sequencing (PhIP-Seq), that allows us to study the infection history of individuals to large numbers of viruses simultaneously. This technology uses bacteriophages to express and display viral antigens of choice, which are then bound by antigen-specific antibodies in patient samples. Antibody-bound bacteriophages are pulled down and identified through molecular techniques. This technology has been used in various infectious disease scenarios, including assessing exposure to different viruses, studying vaccine responses, and identifying viral cause of diseases. Despite inherent limitations in presenting only peptides, this technology holds great promise for future application in identifying novel pathogens, one health and pandemic preparedness.

**Abstract:**

Phage ImmunoPrecipitation Sequencing (PhIP-Seq) is a high throughput serological technology that is revolutionizing the manner in which we track antibody profiles. In this review, we mainly focus on its application to viral infectious diseases. Through the pull-down of patient antibodies using peptide-tile-expressing T7 bacteriophages and detection using next-generation sequencing (NGS), PhIP-Seq allows the determination of antibody repertoires against peptide targets from hundreds of proteins and pathogens. It differs from conventional serological techniques in that PhIP-Seq does not require protein expression and purification. It also allows for the testing of many samples against the whole virome. PhIP-Seq has been successfully applied in many infectious disease investigations concerning seroprevalence, risk factors, time trends, etiology of disease, vaccinology, and emerging pathogens. Despite the inherent limitations of this technology, we foresee the future expansion of PhIP-Seq in both investigative studies and tracking of current, emerging, and novel viruses. Following the review of PhIP-Seq technology, its limitations, and applications, we recommend that PhIP-Seq be integrated into national surveillance programs and be used in conjunction with molecular techniques to support both One Health and pandemic preparedness efforts.

## 1. Introduction

Serology plays an important role in the diagnosis of various disease processes, especially for infectious diseases [1]. In most individuals, infection with a pathogen leads to the production of pathogen-specific antibodies. These antibodies can then be probed to identify the etiology of infection and disease. Various stages of disease and location of infection lead to the generation of different antibody isotypes which provides an additional layer of information. For example, IgM is produced during acute infection [2], IgG is detected systemically long after pathogen clearance [3], while IgA is generated mostly following mucosal infections [4] and IgE is seen in the case of allergies and chronic infections. Detailed information on antibody isotypes and location of detection not only possess diagnostic value, but also provide insight into immunity against re-infection. 

Nucleic acid-based molecular techniques for the detection of infectious pathogens have revolutionized diagnostic medicine. However, their utility is only apparent within a short window for acute infectious diseases and that too if sampled at the correct site. Furthermore, molecular techniques are associated with higher costs and processing time, which are not ideal for low- and middle-income countries (LMIC) settings or rapid diagnosis. In contrast, the parallel use of pathogen-specific antibody detection can be rapid, cheap, and allow the identification of infectious etiology even after the pathogen has been cleared by the immune system [5]. This means that serology allows for the determination of not only current infections but also prior infections.

Conventional serological techniques (Table 1), such as the western blot (WB), immuno-fluorescence assay (IFA), and enzyme-link immunosorbent assay (ELISA) are the backbone of clinical diagnostic and research laboratories [6]. Their applications within infectious diseases are well-established, and many clinical tests are based on these three techniques. However, key limitations of these techniques are their low throughput and the inability for multiplexing tested pathogens. This is unlike modern high throughput, multiplexable chip-based and bead-based assays (Table 1) [7,8], although even these multiplexable assays require the expression and purification of target antigens, which may not be feasible for some pathogen proteins. A newer form of antibody detection assay overcomes these limitations by utilizing phage display for antigen expression and nucleic acid-barcodes for multiplexing. This newer antibody technology is known as phage immunoprecipitation sequencing (PhIP-Seq).

The concept of PhIP-Seq was initially introduced by Larman et al. in 2011 to detect autoantibodies and identify autoantigens in patients with paraneoplastic syndrome, a condition with an autoimmune etiology [9]. This technique leverages on more modern sequencing and nucleic acid synthesis abilities. Since then, its use has expanded to infectious disease applications including sero-epidemiology of common (e.g., measles) and emerging pathogens (e.g., SARS-CoV-2). In this review, we provide an introduction to the technology, with a focus on viral pathogens. We will also provide an overview of the current sero-epidemiological applications and identify future directions where PhIP-Seq’s strengths can be capitalized. 

## 2. Phage Display for Serology prior to PhIP-Seq

Phage display was first described by George Smith in the 1980s for studying protein–protein interactions [10]. Since then, the phage display technology has evolved to be of utility within many other areas of biomedical sciences, including the study of antibodies [11]. Phage display libraries are widely used to both discover novel antigen-specific monoclonal antibodies and also to epitope map existing antibodies [12,13]. However, while phage-display technology for studying antibody responses have been present for several decades, it has often been plagued by several common issues. For example, phage display libraries are often created using random n-mers [14] or cDNA products [15,16], which when created from the target tissue through random priming leads to ORFs of various lengths or premature stop codons [17]. Furthermore, earlier phage display technologies utilize the M13 lysogenic phage display system, which requires antigenic phages to be translocated across the periplasmic membrane, thereby introducing bias against certain amino acid peptide sequences [18]. Fortunately, advances in molecular technologies (such as microarray-based DNA synthesis and high throughput sequencing) and the use of T7 bacteriophage has enabled PhIP-Seq to overcome these challenges [9].

## 3. PhIP-Seq Technology

**Figure 1:**
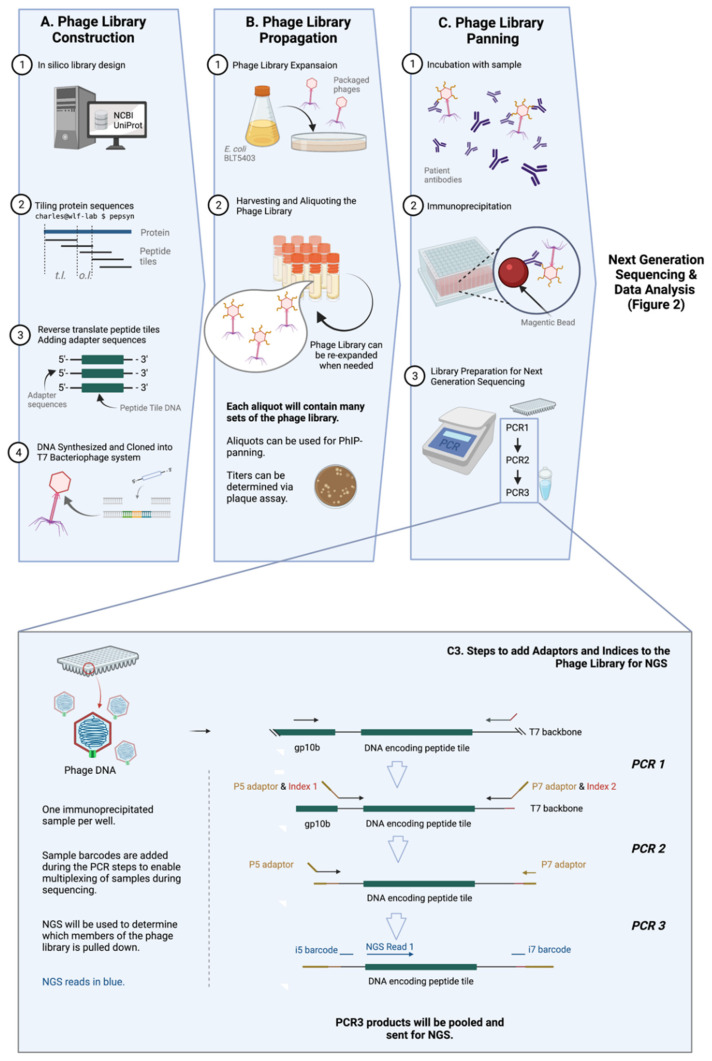
General workflow of PhIP-Seq. The PhIP-Seq methodology is composed of four key steps: (**A**) phage library construction, (**B**) phage library propagation, (**C**) phage library panning, and data analysis (displayed in Figure 2). (**Inset**) Addition of adapters and indices to immunoprecipitated phage sample using PCR (step C3 of Figure 1A is described in detail). Abbreviations: tile length (t.l.), overlap length (o.l.). Created with BioRender.com.

PhIP-Seq is an antigen-specific antibody detection assay that is high throughput, highly sensitive, and mega-plexable (i.e., ability of plexing over a million different peptides). Numerous PhIP-Seq libraries panning different types of proteins have been developed in the recent years. Following the seminal study identifying autoantigens [9], PhIP-Seq has since been expanded to include infectious diseases, such as viruses (including numerous human viruses, arthropod-borne viruses, SARS-CoV-2, etc.) [19,20,21], and bacteria (mostly in the form of human microbiota) [22]. A schematic representation of the typical PhIP-Seq workflow is displayed in Figure 1 [23]. 

### 3.1. Phage Library Construction

Library design is rational [22] and does not require tissue, transcripts, directional cloning, or in-frame ORF determination. Almost any genetic sequence, regardless of its identity and whether it has been previously expressed or purified, can be cloned and tested via PhIP-Seq. This versatility allows for numerous known strains and infectious pathogens (viruses [24,25], and possibly parasites and fungi, etc.), as well as relatively new pathogens to be easily included in PhIP-Seq libraries when available. Libraries containing targets to novel pathogens, like SARS-CoV-2, can be made with relative ease, as seen in Shrock et al. [19] (Figure 1A).

One of the key improvements of PhIP-Seq is its use of an in-silico designed and custom-built library. Unlike traditional phage display, PhIP-Seq’s library is composed of sequences with defined lengths, overlaps, and a known annotation (e.g., 56 amino acid tiles with 28 amino acid overlap, as in the case for the VirScan—one of the earliest PhIP-Seq libraries) [20]. The tools (pepsyn, a Python-based tool that designs peptide libraries) and the shell codes required for the design of these customized libraries are readily available online [23]. Briefly, the set of codes converts the input amino acid sequences (in the fasta file format) to nucleic acid sequences that codes for the corresponding peptide set tile length and tile overlap. The sequences can then be synthesized using a microarray-based DNA synthesis platform, which may then be cloned into a commercially-available T7 phage display system. 

### 3.2. Phage Library Propagation

The PhIP-Seq system utilizes the T7 bacteriophage, a lytic phage, as its powerhouse to express antigens for detection. T7 bacteriophage expresses copies of the target epitope or peptide on its surface, and the identity of this target epitope is reflected in its genomic sequence. PhIP-Seq is composed of ‘live’ phages, i.e., replication-competent phages [26]. Phages (Figure 1B) are expanded within a specified bacterial host grown on solid phase, and progeny phages in supernatant are collected. These progeny phages will carry the same genetic information and therefore, the same corresponding peptide tiles on their surface as the parental phages. Upon expansion, the phage library can be used for further expansion or for PhIP-Seq experiments. 

### 3.3. Phage Immunoprecipitation 

Immunoprecipitation of PhIP-Seq phages with antibodies is typically straightforward and not very different from traditional phage display. Patient or animal [27] sera or cerebrospinal fluid (CSF) (or theoretically, any biological fluid containing antibodies, such as saliva, urine, etc.) can be used to probe PhIP-Seq libraries. Biological samples are typically first measured by ELISA to ensure the presence of antigen-specific immunoglobulin. This is followed by the incubation of antibody-containing sample and the peptide-displaying phages, and then an immunoprecipitation step which typically involves protein A/G on magnetic beads. (Figure 1C) More detailed investigations into isotype- (i.e., IgG, IgA, IgM or IgE, etc.) or subclass (i.e., IgG1, IgG2, IgG3, IgG4 etc.)-specific humoral responses can also be conducted by pulling down using beads coated with the relevant anti-isotype or anti-subclass monoclonal antibody [28] or reagents. For example, in one published study, the authors used streptavidin-coupled magnetic beads with biotin-conjugated omalizumab to pull down and study IgE-specific responses [29]. 

Before the identity of immunoprecipitated phages can then be determined with high throughput sequencing, a few steps of pre-processing are required. This involves amplifying the peptide tile specific sequence of the phage genome by polymerase chain reaction (PCR), subsequently adding sample barcodes and eventually the next generation sequencing (NGS) adaptors, e.g., P5 and P7 (Figure 1C and the inset). Sample barcodes used to identify individual samples as PhIP-Seq runs are typically multiplexed given the read depths of modern NGS systems. 

The high sensitivity provided by using molecular sequencing as the detection method, can lead to some assay background. To account for background signal, each run is typically supplemented with a number of negative controls (i.e., immunoprecipitation of phage library in the presence of PBS only) and library controls (i.e., where only the input library is sequenced). The former captures the background due to direct phage-bead interaction in the absence of antibodies or components from biological samples, while the latter captures the available breadth of the library. Additionally, technical duplicates can also be run for each sample and averaged to improve accuracy [24,30]. In other works, antibody binding to rare or uncommon viruses (such as Rabies and Ebola virus) are used as baseline controls for samples to account for sample-to-sample variations in sequencing depth [31]. Intra-subject comparison of time-course samples (i.e., samples taken from pre- and post-infection) can also be used to account for baseline background signal and can more clearly show the changes in the antibody repertoire brought upon by a specific infection or exposure event [32]. 

### 3.4. Data Analysis Overview 

There is no unified pipeline or script for the analysis of PhIP-Seq data. However, several research groups are trying to establish computational pipelines and make it available online for general use (https://github.com/lasersonlab/phip-stat, accessed on 20 April 2022), (phippery, https://github.com/matsengrp/phippery and phip-flow, https://github.com/matsengrp/phip-flow, both accessed on 20 April 2022). 

**Figure 2:**
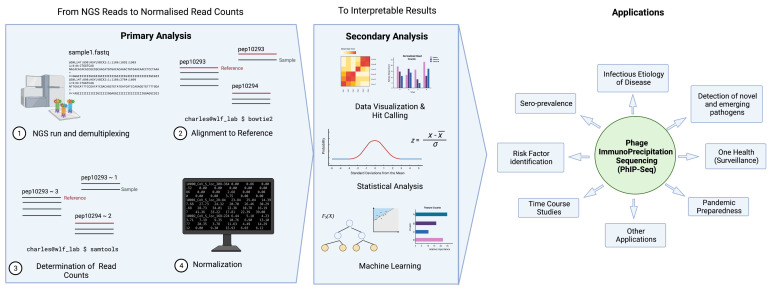
General data analysis pipeline and applications of PhIP-Seq. Created with BioRender.com.

Figure 2 illustrates a typical pipeline of data analysis; here we demonstrate an example used by the pipeline, phip-stat (https://github.com/lasersonlab/phip-stat, accessed on 8 May 2022). High throughput sequencing data is demultiplexed to identify PhIP-Seq data specific to each sample and aligned to reference sequences generated from the original sequence files to deconvolute the peptide IDs. The number of reads for each specific peptide tile is then counted for each sample, which is followed by normalization with the read (either to a set number of reads per sample or using a mathematical model). Typically, normalized read counts will be reported and this can then be used for downstream analysis. Following normalization, some studies will apply additional statistical testing based on poison distribution (and use a *p*-value-based metric (−log10(p)) [20,25,33], or utilize a *z*-score metric to describe enrichment of peptide tiles [31,32,34]). The resulting metrics may then be used to analyze and visualize the phage immunoprecipitation results. 

There are two key steps involved in converting NGS reads to interpretable results. Primary analysis entails data cleaning, FASTQ quality control of NGS data, alignment of reads to reference sequences, assessment of read counts, and data normalization. Depending on the scientific question and application, data will undergo secondary analysis in the form of data visualization, statistical analysis, and machine learning, etc. Created with BioRender.com.

### 3.5. Determining the Hits

Calculating a *z*-score metric or a *p*-value metric may not be sufficiently informative as investigators are typically more interested in the actual significance of the result—i.e., if it indicates a prior exposure or otherwise. 

A key problem with the use of high throughput serology is not having a “gold standard comparator”—many of the target proteins typically will not have commercial serological tests available. Without a standard comparator to allow us to “train” the platform, it will be challenging to determine which *z*-score, or *p*-value would correspond a “true hit” or a prior exposure. Thus, researchers frequently use statistical measures and controls to determine which peptides or viruses are considered positive for a PhIP-Seq run. Cut-offs are sometimes defined, for example, based on a reproducibility threshold based on the −log10(*p*-value) of technical duplicates, and an epitope is considered to be positive when this threshold is crossed [33]. Confidence to the scoring algorithm and cut-offs is typically bolstered with the measures of antibody response to common human infections (e.g., rhinovirus, or Epstein-Barr virus infections), cross-comparison against conventional serological assays, or provided for by an infection history (for example, clinically diagnosed infected patients). Statistical tests such as *t*-tests [24] and the Mann–Whitney test [21,25,33,35], amongst others, are also frequently used to compare data generated from PhIP-Seq.

### 3.6. Other Analysis Strategies (Machine Learning, AVARDA, Novel Pipelines)

More advanced statistical tools are sometimes used to analyze and interpret results from PhIP-Seq. In some studies, authors use gradient boosting algorithm xgboost [36] to determine important peptide tiles (or features) that distinguish one group of patients from another [19,22,33]. This specially works well for case-control studies with large well-defined study populations.

Another involves the use of epitope similarity across different peptide tiles. Monaco et al. attempted to improve the VirScan platform by accounting for possible cross-reactivity among peptide tiles by sequence alignment. This technique, named Antiviral Antibody Response Deconvolution Algorithm (AVARDA), is based on the premise that antibodies can cross-react with similar peptide tiles assigned to different viruses. Antibody cross-reactivity between viruses has not been accounted for in prior PhIP-Seq analysis strategies, and this valuable information is usually lost during analysis [37].

### 3.7. Programming Language and Skills Needed

Primary and secondary data analysis require basic knowledge of command line interface and access to a decent computing infrastructure [23]. Most data analysis can be conducted with Python or R scripts. Having a working knowledge on these programming languages, or having access to individuals who are experienced in data science or bioinformatics, will be of great utility to investigators utilizing PhIP-Seq as a serological tool. 

## 4. Improving PhIP-Seq

While PhIP-Seq used a significantly improved version of phage display technology, inherent technical limitations remain. First, many conformational epitopes will be missed. The library creation step of PhIP-Seq involves the creation of peptide tiles <100 aa in length. This means that although secondary protein structures (such as alpha helices and beta sheets [38]) may be preserved, more complex antibody epitopes [39] that involve protein folding bringing distant regions together or different proteins together will not be recapitulated on the phage surface. Conformational epitopes may be immunodominant for some proteins or pathogens and this may preclude the identification of pathogens that are mainly targeted by conformational antibodies [39]. In fact, in one of the earlier phage display studies, where clones are produced by cloning in ORFs from source tissue (hence different phages will be presenting different length peptide tiles), authors noticed that phages presenting longer peptide tiles typically were more enriched. They postulated that shorter peptide tiles may not contain as much conformational epitopes compared to longer peptide tiles to be enriched [17]. 

Secondly, as PhIP-Seq displays peptide epitopes, humoral responses against non-peptide epitopes, such as carbohydrate antigens, will not be captured. For example, in Vogl et al., where the authors study the antibody repertoire against gut microbiota, they indicated that antigens such as lipopolysaccharides (which are strong modulators of the immune response against bacteria) are not detected by PhIP-Seq [22]. Similarly, as phage displayed peptide tiles are products of bacteria, PhIP-Seq inherently lacks eukaryotic post-translational modifications (PTMs) of proteins and therefore can pose a challenge in cases where PTMs on pathogens are targeted by the humoral immune response. The PTM problem is partially addressed in a recent work where the investigators used a phage library modified by a PTM enzyme, calcium-dependent peptidylarginine deaminase (PAD), to citrullinate relevant peptide tiles [40]. While this proof-of-concept study was successful in improving the detection of citrullinated proteins in patients with rheumatoid arthritis, more work must be performed to cover the numerous other PTM processes in eukaryotic cells.

Thirdly, there still exists some degree of non-specificity in the PhIP-Seq platform. Although each peptide tile is annotated as part of a specific pathogen or protein, an antibody raised against pathogen X, for example, may bind to pathogen Y because both proteins share high sequence similarity. While the AVARDA algorithm attempts to resolve this [37], more work needs to be performed to improve the algorithm and to work around the issue of accounting for mimotopes. Probing large sample sets with PhIP-Seq libraries and investigating different machine learning algorithms may allow for the tackling of non-specificity and recognition of pathogen-specific antibody signatures.

Finally, we would also like to highlight the importance of having a unified (or standard) pipeline for data analysis (which is currently lacking) and a database for the customized PhIP-Seq library with annotation updated. This will improve reproducibility of the data and allow for a cross-experiment and cross-study data comparison and collaborations. Having a mechanism to share data, in the raw and processed form, will also be helpful to promote and expand the use of PhIP-Seq. Depending on the scientific question, only a fraction of PhIP-Seq data is typically used and reported in each study. A common repository or sharing of PhIP-Seq data will enable the utilization of extraneous and unused data to conduct additional investigations. Publishing PhIP-Seq results can be supplemented by a common reporting standard, where the sample source, objective of the study, library used, analysis performed, and (serological) validation conducted (SOLAV, an example is provided in Supplemental Table S1) are provided in a tabular form. Having a common or standardized reporting system, as such, will offer rapid and easy sharing of information about the precise technical characteristics of a PhIP-Seq run and allow for more convenient data sharing focusing on the principles of reproducibility and transparency. 

## 5. Applications of PhIP-Seq in Sero-Epidemiology

PhIP-Seq is increasingly being used to investigate the sero-epidemiology of infectious diseases, especially those caused by viruses. Here, we provide a brief overview of the different applications of PhIP-Seq in epidemiological investigations, such as pathogen exposure, disease etiology, risk factor analysis, temporal kinetics, vaccines, and the identification of novel pathogens in humans and animals (One Health). 

While no means exhaustive, this work mainly outlines the possible applications of PhIP-Seq to the field of sero-epidemiology. We found the technology versatile and can be used to investigate infectious disease seroprevalence, risk factors, time trends, infectious etiology of disease, vaccines, novel and emerging pathogens, and applications for One Health and pandemic preparedness (Figure 2).

### 5.1. Seroprevalence Studies

PhIP-Seq can be used to assess the seroprevalence of pathogens in a population. For example, in Xu et al. [20], the authors attempted to determine exposure to the human virome and its differences according to geography and HIV-status. Employing PhIP-Seq (specifically VirScan), the authors showed that common viruses such as herpesvirus, rhinovirus and adenovirus are frequently detected in healthy populations. Furthermore, the study yielded “public epitopes”, i.e., epitopes commonly targeted by a multitude of individuals. This study highlights PhIP-Seq’s strength in its ability to determine antibody targets at the epitope or peptide level, as well as the extended use of this technology for sero-surveillance of infections to aid in vaccination programmes. 

In a more recent study, Vogl et al. developed a PhIP-Seq library to capture the population-wide diversity of antibody responses to the human gut microbiome. The authors performed metagenomic sequencing on more than 900 stool samples and used the microbiome sequence information to design a novel phage library. In this study, the authors found both individual and public ‘responses’ to microbiota epitopes, including age- and gender-related differences. The modeling of the age- and gender-specific serum repertoires using gradient boosting algorithm revealed that antibody epitope repertoires were more longitudinally stable and hence better in its predictions than using metagenomic data [22].

### 5.2. Risk Factor Analysis and Association Studies

The power of PhIP-Seq lies with its high throughput and mega-plexable nature. This is particularly useful when performing a case-control or case-cohort studies to evaluate how the history of virus exposure impacts a defined clinical outcome. The antibody repertoire of banked specimens or sera for patients with a certain condition can be compared to those of matched controls, and differences identified between the two groups can be subjected to further investigation to identify possible associations and risk factors. For example, Hasan et al. [25] conducted a sero-survey to identify possible associations between infection history and obesity. Using the expanded VirScan library, the authors observed that obesity in adults was associated with greater prevalence of peptide hits linked to herpes viruses, such as EBV, HSV1, and HSV2. Another case-control study utilized PhIP-Seq to identify sero-signatures of oncogenic viruses (such as hepatitis B and C viruses) in at-risk populations that were predictive of hepatocellular carcinoma (HCC) onset (even prior to clinical diagnosis) [33].

### 5.3. Vaccinology and Response to Vaccines

PhIP-Seq can also be used to probe questions around vaccine immunity. For instance, a study used PhIP-Seq to elucidate the impact of measles infection and vaccination on humoral immunity. PhIP-Seq data showed that measles virus infection (assayed by an increase in positive measles epitope hits) led to a noticeable decrease in memory antibody responses against other viruses [24]. Interestingly, a similar diminishing of humoral responses was not observed in measles-vaccinated infants, highlighting the protective effects of measles vaccination against other pathogens [24].

Given the resolution of PhIP-Seq, vaccine-induced antibody responses can also be determined at the epitope level. This may be of utility in vaccinology, especially in the determination of target epitopes by vaccination and how these differ with doses, prime-boost regimes, and innate infection. One such example was highlighted by a recent study where PhIP-Seq revealed that vaccination (as compared to SARS-CoV-2 infections) generated more diverse antibody epitopes [41]. Another study utilized PhIP-Seq epitope mapping data from COVID-19 patients to identify dominant linear B cell epitope regions and then rationally design peptide vaccine candidates against SARS-CoV-2 [42].

### 5.4. Time Course Studies

Temporal changes in infectious disease landscape can easily be probed using PhIP-Seq. Published studies have displayed the versatility of this technology in investigating humoral kinetics to either a single virus or groups of different viruses. For example, a temporal virome-wide study was conducted in hematopoietic cell transplant patients, which led to the finding that although virome-wide antibody repertoires of the recipients mirror that of the donor soon after transplantation, over time, the antibody repertoires revert to the pre-transplantation state [43]. PhIP-Seq is increasingly being applied to study temporal changes of exposure to a specific pathogen. For instance, in the recent study by Eshleman et al., the authors extracted and studied PhIP-Seq data specific to human immunodeficiency virus (HIV). Therefore, through a virus-specific analysis the authors were able to identify HIV peptide hits that predict infection [31]. 

### 5.5. Infectious Etiology of Disease Studies

PhIP-Seq has successfully been applied to ascertain etiology in cases where a patient or a group of patients have an undetermined or unconfirmed diagnosis. Although next-generation sequencing or other PCR-based assays are the techniques of choice for identifying infectious disease etiology, PhIP-Seq can be used to confirm or even identify the etiology in situations when molecular techniques are unable to identify a cause. In a recent study, VirScan (and ELISA) was used to improve the detection of enterovirus A71 infection amongst a pediatric population over nucleic-acid based molecular techniques [35]. In another pediatric study, VirScan was able to identify non-polio enteroviruses as the etiology of acute flaccid myelitis (AFM) even in the absence of a molecular diagnosis [21].

Atypical disease presentations of infections can be challenging to diagnose. In at least one published case, PhIP-Seq successfully identified the infectious etiology of even an atypical disease presentation. PhIP-Seq data displaying the enrichment of dengue virus-specific antibodies in the CSF versus serum identified the etiology of a case of chronic parkinsonism as a neurological dengue infection [34]. Similarly, PhIP-Seq has also been applied to elucidate the infectious etiology of autoimmune diseases. A recent prospective case-control study using PhIP-Seq (and a variety of other serological assays) demonstrated that the risk of developing multiple sclerosis (MS) is significantly higher in cases of EBV infection and even identified EBV peptide hits that can be used as markers for development of MS [32]. Therefore, PhIP-Seq not only provides a tool to elucidate the common infectious etiology, but also can cover atypical conditions and otherwise novel associations. 

### 5.6. Novel and Emerging Pathogens

The versatility and flexibility of PhIP-Seq was demonstrated during the COVID-19 pandemic. The pandemic was caused by SARS-CoV-2, a novel virus member of the SARS-related Coronavirus (SARSr-CoV) family. Given the nature of how the PhIP-Seq library is constructed, having the sequence of novel SARS-CoV-2 and other close coronaviruses (CoV) allows for the quick establishment of a phage-based serological assay. Multiple papers were published describing the application of PhIP-Seq in questions pertaining to SARS-CoV-2 serology. One study described the cross-reactive serological response of SARS-CoV-2 infected patients with that of seasonal, bat, and other severe coronaviruses. They also mapped out immunogenic regions of the SARS-CoV-2 genome (albeit with the limitations of linear peptides), differential analysis of the IgA/IgG response, and attempted to identify correlates of infection severity [19]. Another study used PhIP-Seq to determine the antibody reactivities against SARS-CoV-2 and endemic human coronaviruses and their association with neutralizing antibody titer [44]. Yet another study created a PhIP-Seq library that contains all seven human coronaviruses and 49 animal coronaviruses. Then, with convalescent and pre-pandemic (control) sera, the authors were able to identify reactive and cross-reactive epitopes to various coronaviruses, including animal coronaviruses. The authors claimed that this method of detecting a sero-signature may prove helpful in the early stages of the next pandemic [45].

### 5.7. Applications to One Health and Pandemic Preparedness 

Given the recent trends of zoonotic spillover events, there are increasing efforts to improve infectious disease surveillance at human-animal interfaces [46,47,48]. Since antibody responses last for longer and can be detected even in the absence of nucleic acid or disease, serological techniques (such as PhIP-Seq) should be used in parallel with molecular assays to surveillance for both human pathogens and zoonotic spillover events [49,50]. Furthermore, if used longitudinally, serology detection may be able to identify asymptomatic zoonotic spillovers prior to an epidemic. PhIP-Seq can easily be modified to probe immunoglobulins from different species of animal hosts [51]. For One Health purposes, specialized PhIP-Seq libraries encompassing a wide range of pathogen families, including those deemed as at high risk of causing human disease and zoonosis, should be created. Even if a novel pathogen (of pandemic potential) appears that is not included in the PhIP-seq libraries used for One Health surveillance, the inherent cross-reactive nature of antibody responses and the inclusion of PhIP-Seq peptides from a wide range of pathogen families would allow for the rapid identification of the pathogen type, order, family, or genus, etc. 

## 6. Conclusions

Since the seminal papers describing PhIP-Seq technology in 2011, numerous subsequent studies have been published demonstrating the platform’s ability to be used for both hypothesis generation and testing. Given the level of mega-plexability and the breadth or scope of antigenic targets possible, PhIP-Seq’s applications in sero-epidemiology, diagnostic medicine, and clinical research are broad and far-reaching. Despite the associated cost of NGS, the mega-plexing capability of PhIP-Seq (in terms of both pathogens and samples) allows for a reduced cost per test and increased accessibility for low-resource settings. Going forward, we foresee this technology not only contributing to vaccinology and the sero-prevalence tracking of endemic and emerging infections, but also being used to identify novel pathogens and zoonotic transmission events (i.e., in One Health). We recommend that this technology be developed and used intensively in parallel with molecular techniques as part of national surveillance systems and thereby, be part of pandemic preparedness efforts. With the increasing need for tracking multiple infectious pathogens and the development of global NGS capabilities, PhIP-Seq indeed carries the promise of high throughput serology.

## Figures and Tables

**Table 1:** The table below describes the most commonly used tools to study antibody responses to infectious diseases.

Assay	Solid Phase	Antigen	Detection	Multi-Plexability
ELISA	Plastic plate	Whole proteins or subdomains of interest	Enzyme-tagged antibody	Limited
Western Blot	Nitrocellulose Membrane	Denatured proteins	Enzyme-tagged antibody	Limited
IFA	Cells	Cells expressing protein of interest	Fluorescent-tagged antibody	Limited
Luminex	Beads	Whole proteins or subdomains of interest	Fluorescent-tagged antibody	Medium
PhIP-Seq	T7 Bacteriophage	Peptide tiles	NGS	High

## Supplementary Materials

The following supporting information can be downloaded at: https://www.mdpi.com/article/10.3390/pathogens11050568/s1, Table S1: An example SOLAV statement, with corresponding explanations, for the PhIP-Seq paper by Xu et al., 2015 [20].
