# Supplementary material for: Phage ImmunoPrecipitation Sequencing (PhIP-Seq): The Promise of High Throughput Serology
Source: Pathogens. 2022 May 11;11(5):568. doi: 10.3390/pathogens11050568 (PMC9143919; doi:10.3390/pathogens11050568)
Supplement: Supplementary file 1 [file pathogens-11-00568-s001.zip › pathogens-1715661-supplementary.pdf]

Supplementary Table S1. An example SOLAV statement, with corresponding explanations, for the PhIP-Seq paper by Xu et al, 2015. We hope that the use of SOLAV will allow transparent and efficient sharing of PhIP-Seq results.

**SOLAV Statement**<br>A summary of the PhIP-Seq Experiment Methodology presented in tabular form	
**Description**	**An Example: Xu et al (2015)**
Samples<br>• This refers to the number and type of samples (e.g., serum, CSF, or other sample matrices) used for the experiment.<br>• Positive and negative controls should also be enumerated here, if applicable.	Human sera and plasma samples with different country origins (Thailand, Peru, South Africa, United States). Includes HIV and HCV patients. Total of 569 human samples.
Objectives<br>• This describes the aims of the study – i.e., why PhIP-Seq was used, and the scientific question being answered in the study.	To comprehensively characterize viral-host antibody interactions
Library<br>• This refers to the library used in the study. It may involve citing the source of the library.<br>• If a custom library is made for this study, the authors must include information such as the general composition of the library, tile length and overlaps, positive and negative control peptide tiles, and other modifications to the library (e.g., post-translational modifications).	Viruses with Human Hosts from the UniProt Database, collapsed to 90% sequence identity 56aa tiles, 28aa overlaps, with a library size of 96100 peptide tiles.
Analysis<br>• This includes information on how the data is normalized, and how hits are called.<br>• It is not necessary to indicate the whole pipeline. Authors may also cite previous work.	Zero-inflated Generalized Poisson Distribution to calculate a -log(p-value) score.<br>Hits called when score passes the threshold of reproducibility (described in the article).
Verification<br>• This refers to secondary serological (orthogonal) verification of the results.	Commercial ELISA kits (RSV, Herpesvirus 1 and 2 serology)
